# Supplementary material for: Implemental delay as a mediator of the relationship between depression, anxiety, stress and school burnout
Source: PLoS One. 2024 Dec 31;19(12):e0316082. doi: 10.1371/journal.pone.0316082 (PMC11687754; doi:10.1371/journal.pone.0316082)
Supplement: Attachment — Submitted filename: Response to the reviewer.docx [file pone.0316082.s001.docx]

Katarzyna Markiewicz

WSEI University

Projektowa 4

20-209 Lublin

Dear Editor,

First of all, we would like to thank you and the reviewer for carefully reviewing our paper and for your remarks that made it possible to improve it and make it more readable. We have also taken into account the reviewer's comments and introduced the following amendments, which we marked in blue.

**Response to the first Editor’s letter**

1. Please ensure that your manuscript meets PLOS ONE's style requirements, including those for file naming.

We have prepared the paper in accordance with PLOS ONE's style requirements.

2. When completing the data availability statement of the submission form, you indicated that you will make your data available on acceptance. We strongly recommend all authors decide on a data sharing plan before acceptance, as the process can be lengthy and hold up publication timelines. Please note that, though access restrictions are acceptable now, your entire data will need to be made freely accessible if your manuscript is accepted for publication.

We have indicated that our data are fully available without restriction and included the data availability address so our data will be freely accessible if our manuscript is accepted for publication.

3. A rebuttal letter that responds to each point raised by the academic editor and reviewer(s). You should upload this letter as a separate file labeled 'Response to Reviewers'.

The rebuttal letter was loaded as a separate file after introducing the required amendments.

4. A marked-up copy of your manuscript that highlights changes made to the original version. You should upload this as a separate file labeled 'Revised Manuscript with Track Changes'.

We have uploaded a marked-up copy of our manuscript that highlights changes made to the original version.

5. An unmarked version of your revised paper without tracked changes. You should upload this as a separate file labeled 'Manuscript'.
6. We have uploaded an unmarked copy of our manuscript that highlights changes made to the original version.
7. Please review your reference list to ensure that it is complete and correct. If you have cited papers that have been retracted, please include the rationale for doing so in the manuscript text, or remove these references and replace them with relevant current references. Any changes to the reference list should be mentioned in the rebuttal letter that accompanies your revised manuscript. If you need to cite a retracted article, indicate the article’s retracted status in the References list and also include a citation and full reference for the retraction notice.

We have reviewed our reference list and marked the changes both in the list and in the text. We have added references (number 51 and 53) as the reviewer suggested citations of the studies that support the hypothesis. Since other references from the list were also moved, we have changed the numbers both in the list and in the text.

**Response to the second Editor’s letter**

1. Please amend your Response to Reviewers letter to include a point by point response to each of the points made by the Editor and / or Reviewers. Please follow this link for more information: [http://blogs.PLOS.org/everyone/2011/05/10/how-to-submit-your-revised-manuscript/](http://blogs.plos.org/everyone/2011/05/10/how-to-submit-your-revised-manuscript/)

We have amended our Response to Reviewers letter and included a point by point response to each of the points made by the Editor and Reviewer.

2. Please amend either the title on the online submission form (via Edit Submission) or the title in the manuscript so that they are identical.

We amended the title in the manuscript so the title on the online submission form and the title in the manuscript are now identical.

3. Please ensure that you refer to Table 2 in your text as, if accepted, production will need this reference to link the reader to the Table.

We now refer to Table 2 on page 10 directly after the paragraph in which it was cited.

**Response to the Reviewer**

Dear Reviewer, we thank you once more for your efforts in reading our article and your insightful comments. We have taken them all into account.

1. Abstract: The percentage of female or male participants should be added in the sample description.

The percentage of female participants is added in the sample description of the abstract.

2. The previous literature on the analyzed constructs has been reviewed, but before the Material and method section the need to examine the relationship between them in adolescence should be justified. In addition, the aim and hypothesis that have been written at the beginning of the Material and method section should also appear before this section, that is, at the end of the theoretical review. The hypothesis should include citations of the studies that support it.

We have justified the need to examine the relationship between depression, anxiety, stress and school burnout in adolescence before the Material and method section. We moved the aim and hypothesis before the Material and method section and now they are at the end of the theoretical review. Also, citations of the studies that support the hypothesis are included there.

3. Discussion: This reviewer suggests referring to the study's hypothesis before discussing the results and deepening the practical implications of the results of this research to enrich final considerations.
   We have referred to the study's hypothesis before discussing the results and discussed the practical implications in more detail in order to enrich final considerations.

Sincerely yours,

Katarzyna Markiewicz
